# Feasibility of brain intra-axonal microstructure imaging with ultrahigh B-encoding using MAGNUS ultra-high-performance gradients

**DOI:** 10.1162/IMAG.a.68

**Published:** 2025-07-17

**Authors:** Nastaren Abad, Chitresh Bhushan, Afis Ajala, Tim Sprenger, Luca Marinelli, H. Douglas Morris, J Kevin DeMarco, Maureen Hood, Gail Kohls, Vincent B. Ho, Thomas K.F. Foo

**Affiliations:** GE HealthCare Technology & Innovation Center, Niskayuna, NY, United States; GE HealthCare, Munich, Germany; Uniformed Services University, Bethesda, MD, United States; Walter Reed National Military Medical Center, Bethesda, MD, United States

**Keywords:** high performance gradients, ultra-high diffusion encoding, neuroimaging biomarkers, effective intra-axonal radius, magnetic resonance imaging, brain microstructure, axonal integrity

## Abstract

The MAGNUS high-performance MRI gradient platform delivers G_max_ = 200–300 mT/m, and SR_max_ = 500–750 T/m/s using standard clinical 3.0T system power electronics. This enables the exploration of an expanded diffusion parameter space (b~7–≥30 ms/μm^2^) with reasonable SNR, along with substantially shorter diffusion encoding pulse-widths, echo times, reduced distortion, and blurring from shorter echo spacing. The choice of high b-value diffusion-encoding space can effectively suppress contributions from extra-axonal water, allowing for simplified biophysical models to be explored for non-invasive mapping of intra-axonal content. In this study, the feasibility and reproducibility of mapping *in-vivo* whole-brain effective intra-axonal radius (*r_eff_*), using MAGNUS was assessed. By making use of a test-retest paradigm, reproducibility and sensitivity were evaluated for this new biomarker. Six healthy volunteers were imaged, after obtaining written informed consent, under local IRB-approved protocols with a focus on utilizing the maximum gradient strength of 300 mT/m. Multi-shell dMRI protocols, with a lower bound b = 7 ms/μm^2^ were used for feasibility analysis and short (same-day) and long-term (7-days) test-retest repeatability. To aid in increased precision, a framework for rigorous post-processing incorporating real-valued diffusion data handling and gradient non-linearity correction was integrated. At 300 mT/m, simulations highlight a lower bound threshold for robust detectability of *r_eff_* >1.41 μm. The simulated distribution function was consistent with *in-vivo* measurements, where a mean *r_eff_* = 2.75 ± 0.15 μm was observed for whole-brain white matter (WM) across all volunteers. Left-Right brain white matter asymmetry as a function of *r_eff_* was noted with segmentations of well-reported parcels, such as the corpus callosum and corticospinal tract, demonstrating good agreement with prior literature. Data highlighted good repeatability in voxel-wise and parcel-based estimates for short- and long-term test-retest analysis. A mean coefficient of variance of 3.2% for WM parcels across all volunteers was noted, with a reproducibility coefficient of 0.16 μm (6.6%) highlighting a lack of systemic bias. This study reports on the feasibility of investigating *r_eff_* using MAGNUS. The analysis of repeatability established the floor of changes in the brain that can be observed in studies leveraging *r_eff_* as a neuroimaging biomarker for white matter integrity or for investigating neuroplastic processes in the brain.

## 1 Introduction

Non-invasive quantification of intra-axonal milieu presents an exciting but challenging avenue for gaining greater insight into brain microstructural and functional organization. One of the key features of diffusion weighted MR is its sensitivity to restricted or semi-permeable pore sizes, and thus, mean displacement over fundamental characteristic length scales can be inferred. In white matter (WM), as axonal walls are believed to be the primary hinderance for diffusion (Beaulieu, 2002), the challenge is sensitizing the macroscopic (mm-resolution) diffusion measurement to microscopic (μm-scale) features of axonal morphology. Existing techniques, tailored for the performance of whole-body clinical MR scanners, utilize complex biophysical models to compensate for the low b-encoding space with signal contributions dominated by extra-axonal water (Fieremans et al., 2016; H. H. Lee et al., 2018). However, recent advances in interpretation of biophysical modeling (Dyrby et al., 2013; Fan et al., 2020; Huang et al., 2015; Jones et al., 2018; McNab et al., 2013; Nilsson et al., 2012; Sepehrband et al., 2016; Veraart et al., 2020) as well as increased MR gradient performance (Foo et al., 2020; Huang et al., 2021; Jones et al., 2018) have allowed for a wider diffusion parameter space to be explored with a new class of diffusion MR measurements which are more specific to underlying brain morphology. Specifically, for quantification of the intra-axonal space, several previously confounding factors can be mitigated by leveraging: (a) powder averaging to remove intra-voxel dispersion effects (Anderson, 2005; Callaghan et al., 1979; Jespersen et al., 2013; McKinnon et al., 2017; Mollink et al., 2017; Novikov et al., 2018; Papadakis et al., 1999; Reisert et al., 2017), and (b) gradient hardware capable of producing sufficiently large diffusion weighting (in a short time) to sensitize the signal, allowing for increased sensitivity toward a specific sub-population with suppression of signal from extra-axonal water.

The performance capabilities of next-generation MRI scanners for neuroimaging makes way for higher spectral, spatial, and diffusion resolutions to better elucidate microstructural detail. Imaging with higher gradient amplitudes (>80 mT/m) and slew rates (>200 T/m/s) are a fundamental requirement for mapping information at length scales of 1–10 μm (Jones et al., 2018). To achieve sensitivity over a broad range of macro- and microscopic length scales with high (power-efficient) gradient performance, head-only asymmetric gradients are required (Alsop & Connick, 1996). The reduced design field-of-view (FOV) and inner diameter reduces gradient inductance, while the smaller coil extents significantly reduce peripheral nerve stimulation, demonstrating higher coil efficiencies compared to conventional whole-body systems (In et al., 2020; Lee & Bernstein, 2022; S. K. Lee et al., 2016). For dMRI, strong diffusion encoding (b > 7 ms/μm^2^), shorter diffusion encoding pulse-widths (δ) and mixing times (△), shorter TE times, reduced distortion, and blurring from shorter EPI echo spacing can be achieved (Supplementary Fig. S7). These capabilities have enabled *in-vivo* implementations for higher spatial and angular resolution in tensor and kurtosis metrics, time-dependent diffusivity, and allow for ultra-high b-value diffusion encoding to simplify modeling of intra-axonal diffusivity (Foo et al., 2020; Huang et al., 2021; Tan et al., 2020).

In this study, the head-only MAGNUS (Mesoscale Anatomy Gradients for Neuroimaging with Ultrafast Scanning) 3.0-T MRI (Foo et al., 2020) was used to demonstrate the feasibility of mapping effective axonal radii, *r_eff_*, with ultra-high diffusion encoding. To circumvent the limitations of Rician noise bias and for increased accuracy, decorrelated phase filtering (Sprenger et al., 2017) was used for data pre-processing, along with gradient non-linearity corrections (Tan et al., 2013) for diffusion encoding. Sensitivity for whole-brain characterization of the effective axonal radius was evaluated via a short- and long-term paradigm for test-retest repeatability and reproducibility.

## 2 Theory and Methods

### 2.1 The effective intra-axonal radius

The diffusion parameter space, and approximate axonal diffusion properties were simulated using framework presented by Callaghan et al. (1979) and Novikov et al. (2014) for approximating a system of cylinders with no relaxing walls (impermeable walls / fully restricted cylinder) using the pulsed gradient spin-echo (PGSE) sequence. In the absence of extra-axonal water—assumed to be fully suppressed at high diffusion weighting—this model can be used to yield the intra-axonal, rotationally invariant signal decay as a power-law function, $\bar{S}=\beta b^{-\frac{1}{2}}$ where β is a function of the axonal water fraction and parallel intra-axonal diffusivity and $b^{-\frac{1}{2}}\;$ originates from the 1-D stick model and is a function of diffusion weighting. Recently, studies demonstrated that this power-law relationship suggests vanishing diffusivity perpendicular to the fiber direction, but that in the presence of non-zero intra-axonal radial diffusivity at ultra-high b-values, the signal attenuation deviates from power law scaling allowing for sensitivity to the intra-axonal space (McKinnon et al., 2017; Veraart et al., 2020). To estimate the intra-axonal radial diffusivity and radii, in the long pulse limit, when $\delta \gg \frac{r^{2}}{D_{0}}$, where *D_0_* is the intrinsic diffusivity of the axoplasm, the dependence on Δ, the diffusion mixing time, is negated (Neuman limit) (Neuman, 1974). As such, signal attenuation, or more specifically the orientationally invariant signal attenuation, relates the intra-axonal radial diffusivity and radius as, $\ln\;S^{⊥}\left(r\right)=-\kappa \;r^{4},\;\kappa =\frac{7}{96}\;\frac{g^{2}2\delta }{D_{0}}$ (Burcaw et al., 2015; Veraart et al., 2020). As noted by Veraart et al. (2020), the axonal signal attenuation scales quadratically with its radius *r* demonstrating sensitivity of the dMRI signal to its higher order moments (Veraart et al., 2020).

This framework was used in simulations to assess the accuracy of the approach, and experimental sensitivity to underlying axonal distributions, for realistic experimental parameters accessible in the present study and with two configurations for MAGNUS without gradient re-design. The open-source Microstructure Imaging Sequence Simulation Toolbox (MISST) (Drobnjak et al., 2010, 2011, 2016; Ianuş et al., 2016; Ianus et al., 2013), in MATLAB®, was leveraged to adopt matrix formalism for diffusion signal attenuation within impermeable walls or fully restricted cylinders (Callaghan et al., 1979). A realistic experimental setup for human *in vivo* scans was mimicked, with the parameter space available on MAGNUS with 1-MVA and 2-MVA standard clinical gradient drivers. Simulations were performed for the two system configurations: (1) Gradient strength = 300 mT/m, gradient slew-rate = 750 T/m/s, diffusion time (Δ) = 26.8 ms, gradient duration (δ) = 18.8 ms for the maximum b-value used = 30 ms/μm^2^ and (2) Gradient strength = 200 mT/m, gradient slew-rate = 500 T/m/s, diffusion time (Δ) = 31.3 ms, gradient duration (δ) = 21.4 ms for the maximum b-value used = 30 ms/μm^2^. For consistency with experimental conditions, only PGSE waveforms were considered (other waveforms such as oscillating gradient, double diffusion, and spherical encoding were not in scope). For the simulations, a lower bound b-value of 7 ms/μm^2^ and an upper bound b-value of 30 ms/μm^2^ was used in increments of 0.5 ms/μm^2^, minimizing the likelihood of free water and extra-axonal contributions for finite gradient pulses. To simulate axonal milieu, gamma distributions of cylinders to model restrictions in white matter with a range of diameters spanning 0.5 to 10 μm were used. Histological studies of white matter (Aboitiz et al., 1992; Liewald et al., 2014; Sepehrband et al., 2016) have shown that axon diameters are not uniformly distributed but instead follow a positively skewed distribution, with many small-diameter axons and fewer large ones. The gamma distribution is, therefore, well-suited for representing distributions of axon diameters within a voxel. In line with prior literature (Fan et al., 2020; Nilsson et al., 2017; Veraart et al., 2020), an intrinsic diffusivity D_0_ = 2.0 μm^2^/ms was assumed which is typically used for tissue at 37°C and close to the parallel diffusivity associated with the intra-axonal compartments in well-aligned WM bundles. As complex (real-valued) data were experimentally used, Gaussian noise was assumed, with a minimal SNR ϵ {15-50}. The theoretical lower bound (Nilsson et al., 2017) was further estimated using a single shell at b = 30 ms/μm^2^, and SNR = 20, to determine the feasibility of mapping effective intra-axonal radii with MAGNUS.

### 2.2 MRI experiments

All volunteers were scanned under IRB-approved protocols after obtaining written informed consent. A total of six healthy volunteers were recruited for this study (five males and one female, 47.8 ± 10.8 years). All MR scans sessions were conducted on a MAGNUS head gradient coil installed in a whole-body 3.0T scanner (GE HealthCare, Waukesha, WI). MAGNUS is capable of delivering G_max_ = 300 mT/m and SR_max_ = 750 T/m/s using standard GE HealthCare 3.0 T system power electronics (Signa Premier XT, GE HealthCare, Waukesha, USA) of 2 MVA peak driver power per axis. For this study, the full performance of the gradient system was used to achieve maximum b-value of 30 ms/μm^2^ with a parameter space characterized by Δ/δ = 26.8/18.8 ms and a TE = 56.8 ms. A 32-channel phased array head coil (NOVA Medical, Wilmington, MA, USA) was used for all experiments. For all scans, a multi-shell dMRI protocol (inner shells scaled with respect to b_max_ = 30 ms/μm^2^ ensuring constant gradient duration and separation to exclude time dependency as a confound) was prescribed with axial scan plane orientation and readout set along the Right/Left direction. A 21-cm field-of-view (FOV) with a 96 × 96 acquisition matrix, ±250 kHz readout bandwidth and a slice thickness of 2.2 mm, allowed for 60 slices (whole-brain coverage) with a TR of 5500 ms. Other imaging parameters were: EPI echo spacing = 320 μs, 1 signal average (NEX = 1), R = 2 in-plane acceleration. No slice acceleration was used in this study.

To test the *in-vivo* feasibility of the approach, 60 directions were uniformly sampled on a sphere for 7 b-values (b = 7, 11, 13, 16, 21, 25, 30 ms/μm^2^) with eight interspersed b = 0 scans. This resulted in a total scan time of 41 min. To test the *in-vivo* test-retest repeatability of this approach, five healthy volunteers were scanned twice with one volunteer scanned x3 for determining short (same day) and long (7 days) term repeatability. Herein, an abbreviated multi-shell protocol was utilized with a total of 4 (b = 7, 18, 25, 30 ms/μm^2^) x 60 (n_dir_) encoding directions, with five interspersed b = 0 scans, for a total scan time of 21 min. A lower-bound b-value of 7 ms/μm^2^ was used to minimize residual extra-axonal signal contributions. All other scan parameters were kept identical.

T1-weighted structural images with 1-mm isotropic resolution were also acquired using 3D-MPRAGE for joint spatial normalization and white matter segmentation, with the following imaging parameters: TR/TE = 5400/2.4 ms, inversion prep time = 900 ms, Recovery Time = 1000 ms, flip angle = 8°, for a total acquisition time of 5 min.

No specialized hardware was used for asymmetric gradient concomitant field offsets (Abad et al., 2023) or eddy current correction other than vendor implemented pre-emphasis and integrated spatial gradient non-linearity corrections.

### 2.3 Signal processing and statistical analysis

All diffusion data were processed and fit using a custom in-house reconstruction pipeline (Fig. 4).

#### 2.3.1 Noise mitigation with real valued data

Magnitude reconstruction of the dMRI images results in a rectification of the noise floor (non-central χ^2^ noise is Rician with complex coil combination), resulting in errant estimation for model fitting due to overestimation of the signals amplitude, and reduced image contrast due to the superposition of image signal and noise floor (Fig. 4 & Supplementary Fig. S1). Herein, we adopted decorrelated phase filtering—a kernel-based phase correction technique optimized via the spatial noise correlation patterns (Sprenger et al., 2017). As highlighted by Sprenger et al., optimizing kernel filter design takes into account the propagation of noise in the k-space MRI signal in typical reconstruction pipelines, and relaxes the smoothness requirements on the phase while maximizing the efficiency of the bias correction (effectiveness depends on the correlation of estimated phase with measured noise). The complex conjugate of the filtered phase allows for the rectification of signal into the real domain, and noise in the imaginary domain. In this work, an optimized filter with a kernel size of 3 was used. The output: real-valued data (RVD) that retains zero mean Gaussian noise distribution, which is particularly advantageous for approaches targeting spherical mean/powder-average estimation. In this study, this was a critical step for mitigating Rician bias and extends beyond simple parameter estimation where expectation value tuning is often empirical.

#### 2.3.2 Distortion and gradient non-linearity correction for diffusion

RVD was subsequently corrected for eddy current distortion, bulk motion, and susceptibility. In short, head motion and linear eddy current corrections were performed by means of affine image registration of each diffusion-encoded volume to the non-diffusion encoded image (b = 0/T_2_-weighted), with constraints that it can only apply shear along the phase-encoding direction of the EPI acquisition. Susceptibility-induced EPI distortions were corrected by estimating the EPI distortion field through a phase-encoding constrained cubic b-spline based non-rigid registration of the mean b = 0 image and the undistorted T1-weighted image (Bhushan et al., 2012, 2015). The non-rigid registration framework uses a contrast neutralization approach, named INVERSION, that leverages approximately inverted contrast relationship between T1- and T2-weighted brain MRI images to obtain a robust similarity metric (Bhushan et al., 2015). The estimated distortion-field was used to unwrap the distorted EPI images following the physics of EPI distortion, that is, intensities in un-wrapped EPI images are also modulated by the Jacobian of the transform (Bhushan et al., 2012, 2015). Our image registration framework (rigid, affine, and non-rigid) is implemented using custom components in elastix (Klein et al., 2010).

Distortion-corrected diffusion data were further corrected for spatial non-linearity of diffusion-encoding gradients (GNL) using a custom in-house reconstruction pipeline which allows for the b-matrix to be tracked spatially and temporally (Newitt et al., 2015; Tan et al., 2013). The impact of gradient non-linearity on dMRI is non-negligible in clinical WB systems as well as in asymmetric head only gradient systems where the imaging FOV approaches the design FOV (Malyarenko et al., 2014; Rudrapatna et al., 2021). Since deviations from the desired gradients scale the diffusion attenuation factor quadratically, errors are large. Typical gradient non-linearities observed with the MAGNUS gradients are highlighted in Supplementary Figure S2, with imaging features furthest from isocenter experiencing significant differences in gradient amplitude (>15%). Moreover, it is important to note that segment-based correction and/or b-value scaling alone are not sufficient to account for the errors introduced due to the rotation of the gradient vectors (Supplementary Fig. S3). In this study, GNL effects on diffusion encoding were corrected by first estimating the actual gradient field of each logical gradient axis using the 10^th^ order spherical harmonic expansion. With the estimated field, the temporal evolution of voxel-wise b-value and b-vector deviations was tracked (and adjusted) for parametric fitting.

#### 2.3.3 Spherical harmonic representation for powder averaging

Spherical harmonic (SH) representation for the data was required to extract the rotationally invariant signal features. As highlighted by Bhushan et al. (2014) and Descouteaux et al. (2007), it is assumed that diffusion data are sampled on the surface of the sphere along *q* different diffusion gradient orientations, which are defined in terms of the standard polar angles, $\left(\theta_{q}\;\%\;\left[0\;\pi \right],\varphi_{q}\%\;\left[0\;2\pi \right]\right)$ for *q* = 1,2,… As with all data sampled on the sphere, it is possible to represent the signal matrix for the voxel (v) and *q*-space (s) using an orthonormal basis of SHs which are real and symmetric. The observed/distortion free diffusion signal can be defined by, $S_{v,q}=\sum_{n=1}^{N}C_{v,n}Y_{n,q}$ , where S_v,q_ is the diffusion signal corresponding to the *q*^th^ direction (observed/distortion free); *Y_n,q_* is the *n*_th_ Spherical harmonic basis function sampled along direction *q* (known); and *C_v,n_* is the spherical harmonic coefficient corresponding to the nth spherical harmonic basis function (to be fit). After *C_v,n_* is determined for the spherical mean, coefficients other than the zeroth order are dropped. The spherically averaged signal is then estimated as a dot product per b-value.

#### 2.3.4 Signal fit

To estimate the intra-axonal radial diffusivity and radii in the long pulse limit, representing $S^{⊥}\left(r\right)=-e^{-bD_{a}^{⊥}}$, the effective intra-axonal radius (Veraart et al., 2020) is $r_{eff}={\left((\frac{48}{7}\;\delta \left(\Delta -\frac{\delta }{3}\right)D_{0}D_{a}^{⊥}\right)}^{\frac{1}{4}}$, which was estimated by voxel-wise fits of the real-valued, distortion free, rotationally invariant diffusion signal using a nonlinear least-squares estimator. As noted (Burcaw et al., 2015; Veraart et al., 2020), the attenuation is proportional to the fourth power of the radius *r* and, as such, it is very weak for small axons with the volume weighting emphasizing the thickest axons.

#### 2.3.5 Statistical analysis

For statistical analysis, whole brain white matter (WM), gray matter (GM), and cerebrospinal fluid (CSF) segmentations were obtained using posterior probability maps. To avoid partial volume effects, a probability coefficient of >0.55 for WM, 0.8 for GM, and >0.9 for CSF was used. Johns Hopkins University-International Consortium for Brain Mapping (ICBM)-DTI-81 white matter parcel atlas (Mori et al., 2008) was used for parcel registration to subject space. To evaluate repeatability, WM parcel wise correlation between test-retest was computed across all pairs of repeated observations. Further, Bland-Altman, the Coefficient of Variation (CoV), and Intraclass Correlation (ICC) were estimated per subject over WM for voxel-wise and parcel-based analysis along with spearman’s rank correlation coefficient. Test-retest precision was estimated using the repeatability coefficient with an expectation value of 95%.

## 3 Results

**Fig. 1.**
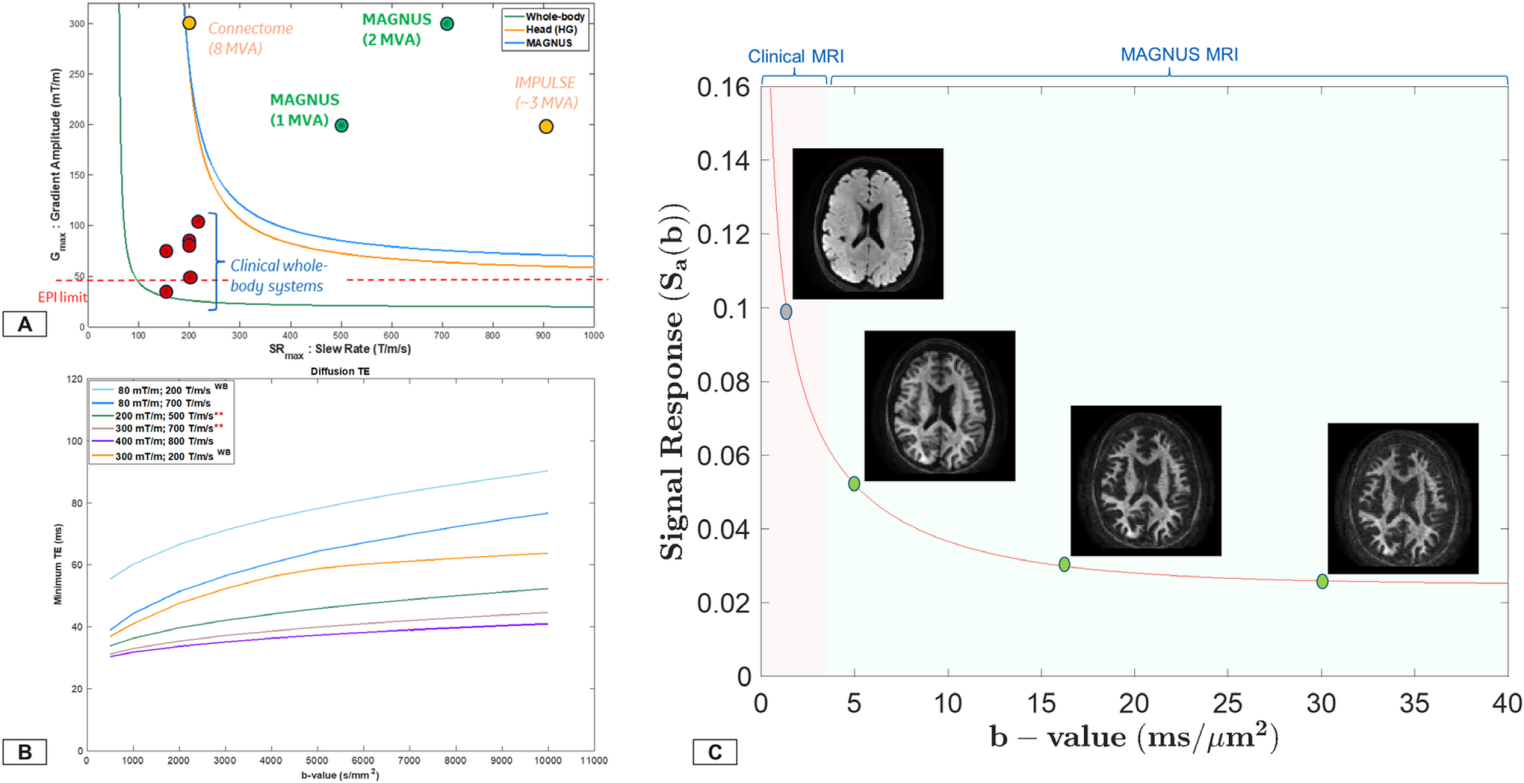
(A) PNS threshold as a function of maximum gradient amplitude G_max_ and SR_max_ for conventional whole-body systems and current state-of-art high performance systems is highlighted for an EPI acquisition with ~50 mT/m readout gradients. Head-only gradients are able to maintain the full performance specifications. (B) Minimum achievable echo-times as a function of maximum diffusion encoding is presented. Unsurprisingly for a maximum b-value of 10 ms/μm^2^, the TE advantage is >2.5x for MAGNUS compared to conventional whole-body systems and is ~1.2x between 200 mT/m and 300 mT/m. As is evident, the TE advantage shows diminishing returns for PGSE acquisitions comparing gradient performance of 300 mT/m versus 400 mT/m. Both A and B highlight the increased SNR and reduced distortion space for DWI (Supplementary Fig. S7). (C) Signal decay as a function of diffusion encoding highlights the expanded diffusion encoding space allowed by high-performance, head-only gradient systems compared to conventional clinical wide bore. Inserts highlight experimental *in vivo* human spherical mean signal to demonstrate attenuation in gray and white matter as a function of diffusion weighting*.* Abbreviations: PNS, Peripheral Nerve Stimulation; G_max_, maximum gradient amplitude (mT/m); SR_max_, maximum slew (T/m/s), MAGNUS, Microstructural Anatomy Gradient for Neuroimaging with Ultrafast Scanning.

**Fig. 2.**
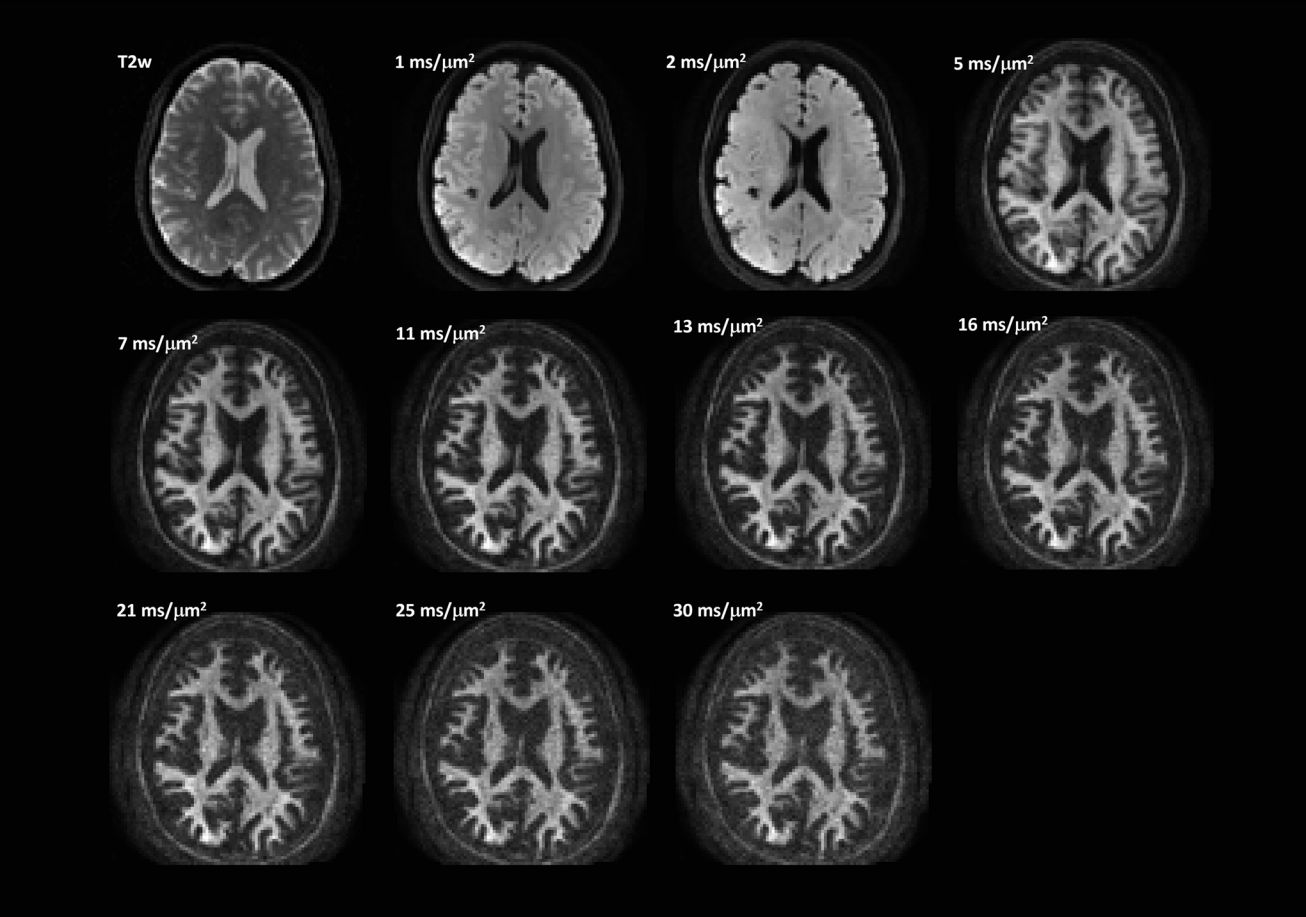
Representative axial scan plane images show the T_2w_, and spherical mean diffusion weighted images presented over an array of b-values for the contrast space utilized in this study. High signal conspicuity and contrast is evident even at ultra-high b-values >20 ms/μm^2^.

The expanded diffusion encoding space highlights striking microstructural detail even at b-values of 30 ms/μm^2^, a regime typically characterized by low SNR (Figs. 1 and 2). With high-performance gradient systems, such as MAGNUS, there is improved acoustic noise performance and mitigation of diffusion-encoding blur due to the more than 2x shorter TEs (Fig. 1B) and echo-planar imaging (EPI) echo spacing achieved with MAGNUS compared to that of whole-body 3.0 T systems.

**Fig. 3.**
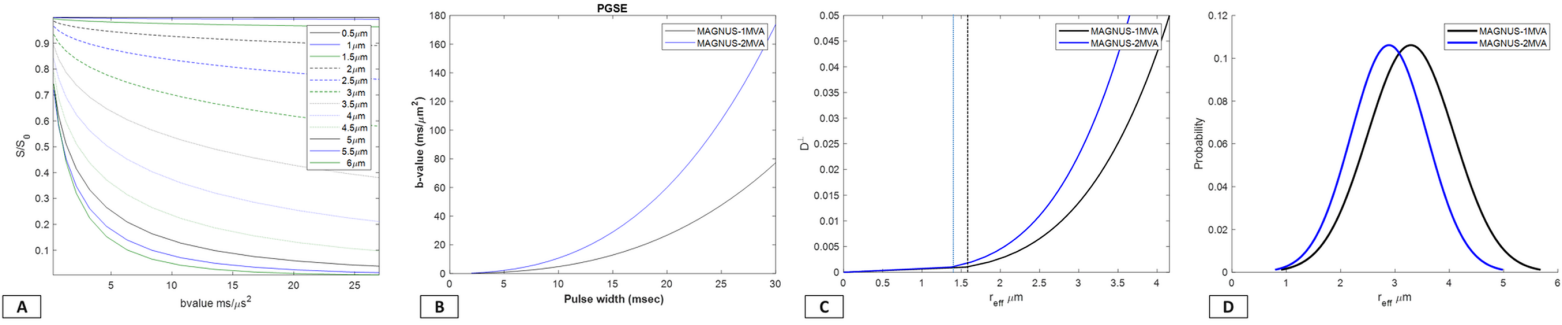
Modeling axons as gamma distributed impermeable cylinders, simulations illustrate the impact of MR gradient performance on imaging performance and content retrieval. By utilizing realistic scan parameters available with two configurations for MAGNUS—without gradient re-design—by switching the gradient driver from 1 MVA to 2 MVA. (A) Highlights signal attenuation as a function of the applied diffusion weighting for a range of axonal diameters for PGSE acquisitions. (B) Plots highlight the pulse duration that is accessible as a function of diffusion encoding across the two configurations. (C) Plots highlight the change in radial intra-axonal diffusivity *versus* the effective radius for two current research configurations of MAGNUS at 1-MVA with a G_max_ of 200 mT/m and at 2-MVA with a G_max_ of 300 mT/m respectively. Data presented assume a maximum diffusion encoding of b = 30 ms/μm^2^ with Gaussian noise and an SNR of 20. Line plots demarcate the minimum observable effective radius of 1.65 μm (black) and 1.4 μm (blue) adopting the formalism for minimal detectability in cylindrical radii (Nilsson et al., 2017). (D) The distribution of effective intra-axonal radii estimated via $\ln S^{⊥}\left(r\right)=-\kappa \;r^{4},\;\kappa =\frac{7}{96}\;\frac{g^{2}2\delta }{D_{0}}$ for both scanner configurations are highlighted.

Simulations reinforce the expected trend of decreasing signal attenuation with smaller apparent radii, that is, there is less attenuation in the signals arising from small diameter cylinders (Fig. 3). The simulation results place the bounds on the resolution limit (defined Nilsson et al. (2017) as cylinders with a diameter below the resolution limit and indistinguishable from cylinders with a diameter of zero) of the current acquisition ~1.4–1.5 μm, thereby suggesting lack of sensitivity to reliably distinguish axon diameters below this threshold. It is inaccurate to consider the resolution limit as fixed, and with increasing gradient performance, unsurprisingly, lower detectability limits can be realized as the echo time, encoding pulse width and the width of the convolution kernel with the ensemble average propagator are impacted. Prior studies (Drobnjak et al., 2016; Dyrby et al., 2011; Nilsson et al., 2017) have estimated a similar 1.5–2 μm range for dMRI-derived measures.

**Fig. 4.**
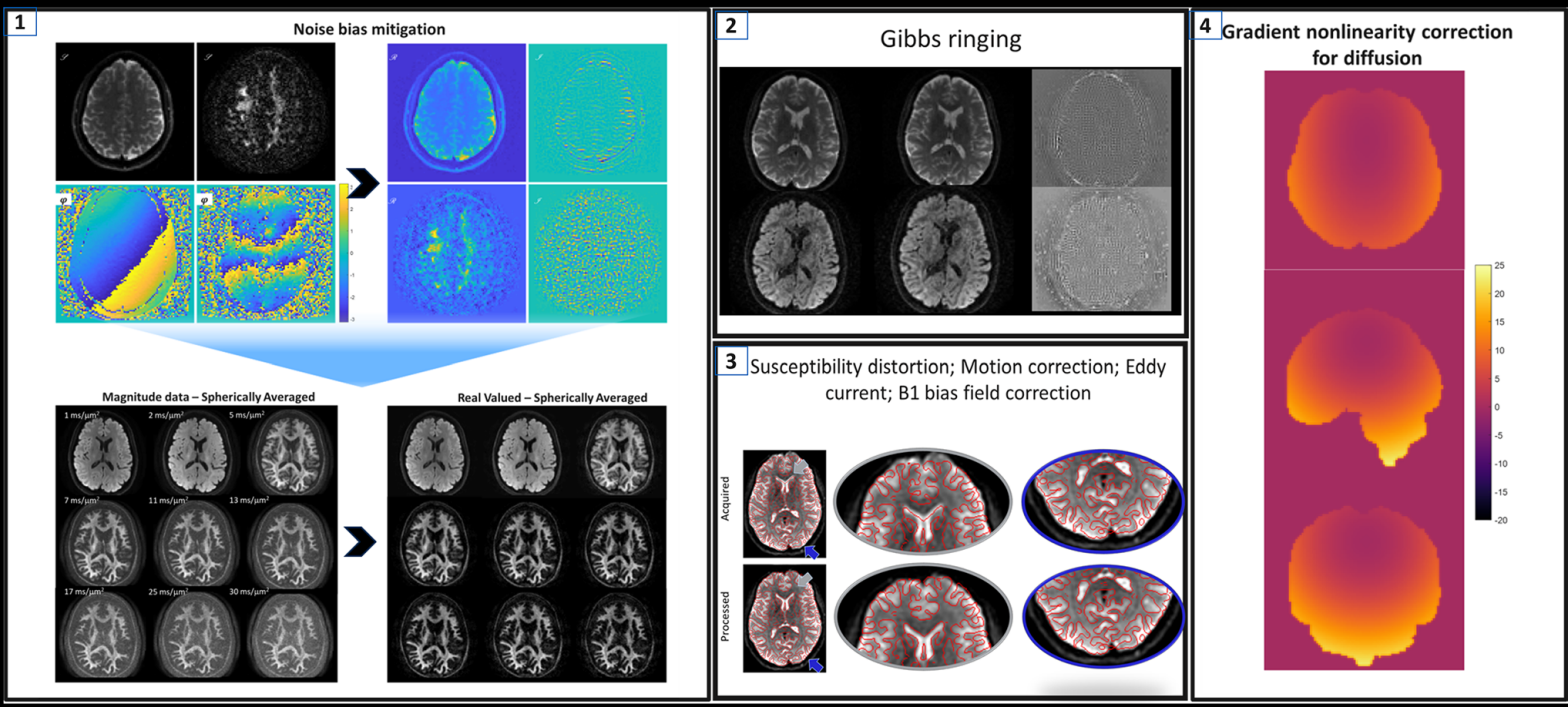
Overview of dMRI processing pipeline used in this study. The first step of the pipeline consists of decorrelated phase filtering for dMRI data, which allows for correlated noise/noise distribution bias to be corrected, with real valued data as an output—circumventing Rician bias. Real valued data are then corrected for between volume motion correction, eddy current-induced distortion correction, bias field correction, and gradient non-linearity correction for the diffusion space before fitting the signal attenuation to estimate effective radius maps (mm). The numbers identify the flow of information through the signal processing pipeline.

In *in-vivo* experiments, reduced contrast due to the superposition of signal and the noise floor was evident in non-denoised data (Fig. 4 & Supplementary Fig. S2), which can adversely affect the signal fit. The non-zero mean noise floor attributed to Rician noise was visually discernable in the magnitude spherical mean data for b > 5 ms/μm^2^_._ Though the likelihood of phase correction errors increases at ultra-high b-values due to increased coherent motion encoding which results in increased sensitivity to strong local phase variations (Supplementary Fig. S6), increased contrast, microstructural detail, and mean noise floor reduction were evident with RVD compared to the non-zero mean noise floor data.

**Fig. 5.**
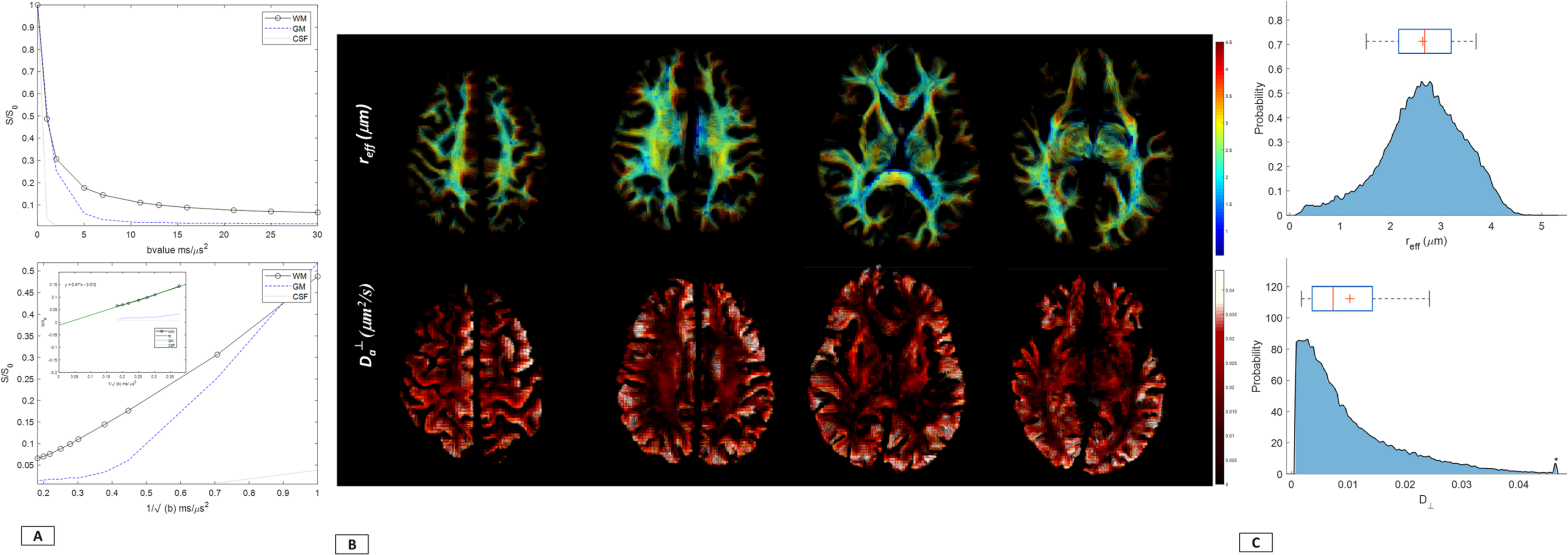
(A) Scatter plots highlight signal decay from whole-brain white matter, gray matter, and CSF segmentation from a (single subject) *in-vivo* human brain both as a function of b-values and as a function of 1/√b. Signal attenuation in white-matter ROIs highlights deviation from the power law scaling in mean white matter (opposite of trend observed in to mean gray matter and cerebrospinal fluid signal), demonstrating sensitivity of the signal to the radial intra-axonal signal for data acquired with the MAGNUS scanner with negative intercept indicating non-zero D _a_^┴^. (B) Representative maps of *r_eff_* (μm) and D _a_^┴^ from a single subject are presented. Maps were overlaid on white matter tractograms with the renderings visualized using Imeka Solutions Inc. (C) Histogram over whole-brain white matter further represent the *r_eff_* distribution observed *in-vivo*. Abbreviations: *r_eff_,* effective intra-axonal radius*;* D _a_^┴^, radial intra-axonal diffusivity.

Signal attenuation over mean whole-brain WM, cortical GM, and CSF up to b-encoding of 30 ms/μm^2^ is shown in Figure 5A, highlighting sensitivity to the underlying microstructural distributions. Notably, extrapolation to infinite diffusion encoding demonstrated a negative intercept with global WM (Fig. 5A). Representative slices from a single subject highlight the dynamic range of distribution for *r_eff_* and *D_a_*^┴^ in *in-vivo* human brain. In these maps, the anatomy of the corticospinal tract, as it projects into the precentral gyrus, the corpus callosum, and the cingulum bundles, is clearly visible and the dynamic range over their corresponding pathlengths can be followed. Furthermore, whole-brain single-subject WM *r_eff_* and $D_{a}^{⊥}\;$ distributions (Fig. 5B-C) demonstrate broad agreement with literature (Barakovic et al., 2023; Fan et al., 2020; Veraart et al., 2020, 2021). In line with the prior literature (Assaf et al., 2008; Barakovic et al., 2023; Fan et al., 2020; Veraart et al., 2020, 2021), the corticospinal tracts exhibited the largest fraction of axons with a diameter greater than 3μm.

**Fig. 6.**
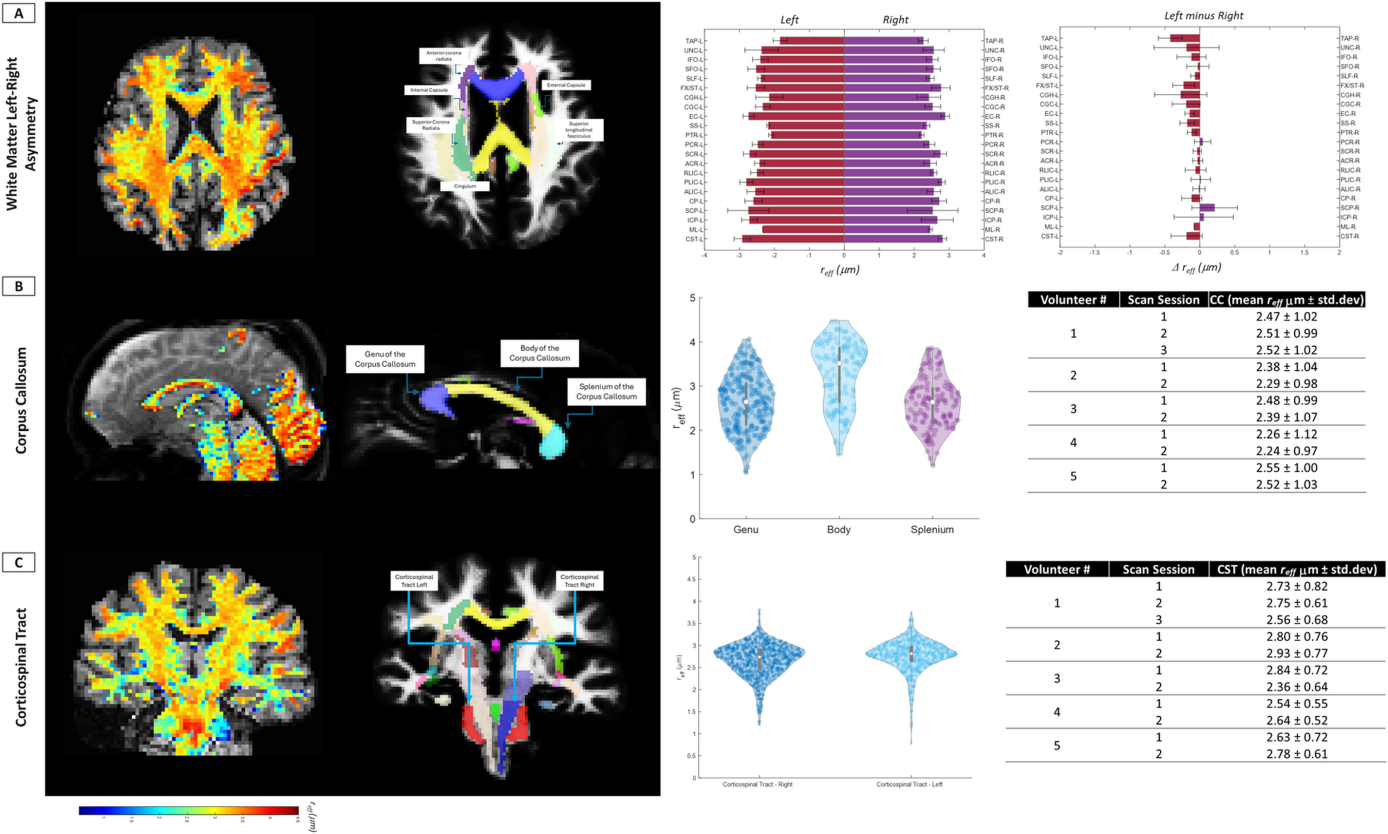
The first column highlights representative effective radius maps from a single subject. Row (A) Descriptive statistics (mean ± std.dev) for brain white matter left-right asymmetry plots were generated using the JHU ICBM WM atlas, for all the volunteers, to highlight the ability to discern microstructural hemispheric variations in the brain. Row (B & C) Representative images from a single subject highlights the dynamic range for *r_eff_* observed for two white matter parcels: (B) the corpus callosum and, (C) the corticospinal tract. Segmentations and associated violin plots highlight the intra-parcel distributions across all volunteers, for *r_eff_,* well within the range reported in prior literature. Tables further subcategorize these descriptive statistics for *r_eff_* for all volunteers, across all scan sessions, evaluated in this study. Abbreviations: *r_eff_,* effective intra-axonal radius*;* CC, corpus callosum; CST, corticospinal tract; std.dev, standard deviation.

Right-Left subdivisions for major white matter parcels allowed for microstructural hemispheric differences, and brain white matter asymmetry, to be identified. The mean parcel asymmetry over all the volunteers in this study is presented in Figure 6A. These representations can potentially be tracked longitudinally to reflect delta-change as a function of pathology or reinforcement of tracks with learning paradigms. Finer segmentations of two white matter bundles, the corpus callosum, and the corticospinal tract are further presented to evaluate inter-bundle variability (Fig. 6B, C), and discernable patterns of variation through WM are evident in the representative images. The violin plots highlight density distributions across the corpus callosum and the corticospinal tracts within the parcellated ROIs for all volunteers. Qualitative and quantitative data are in good agreement with published literature (Fan et al., 2020; Veraart et al., 2021), and associated tables capture the mean distribution for the same parcels for each volunteer recruited for this study (Fig. 6B, C).

**Fig. 7.**
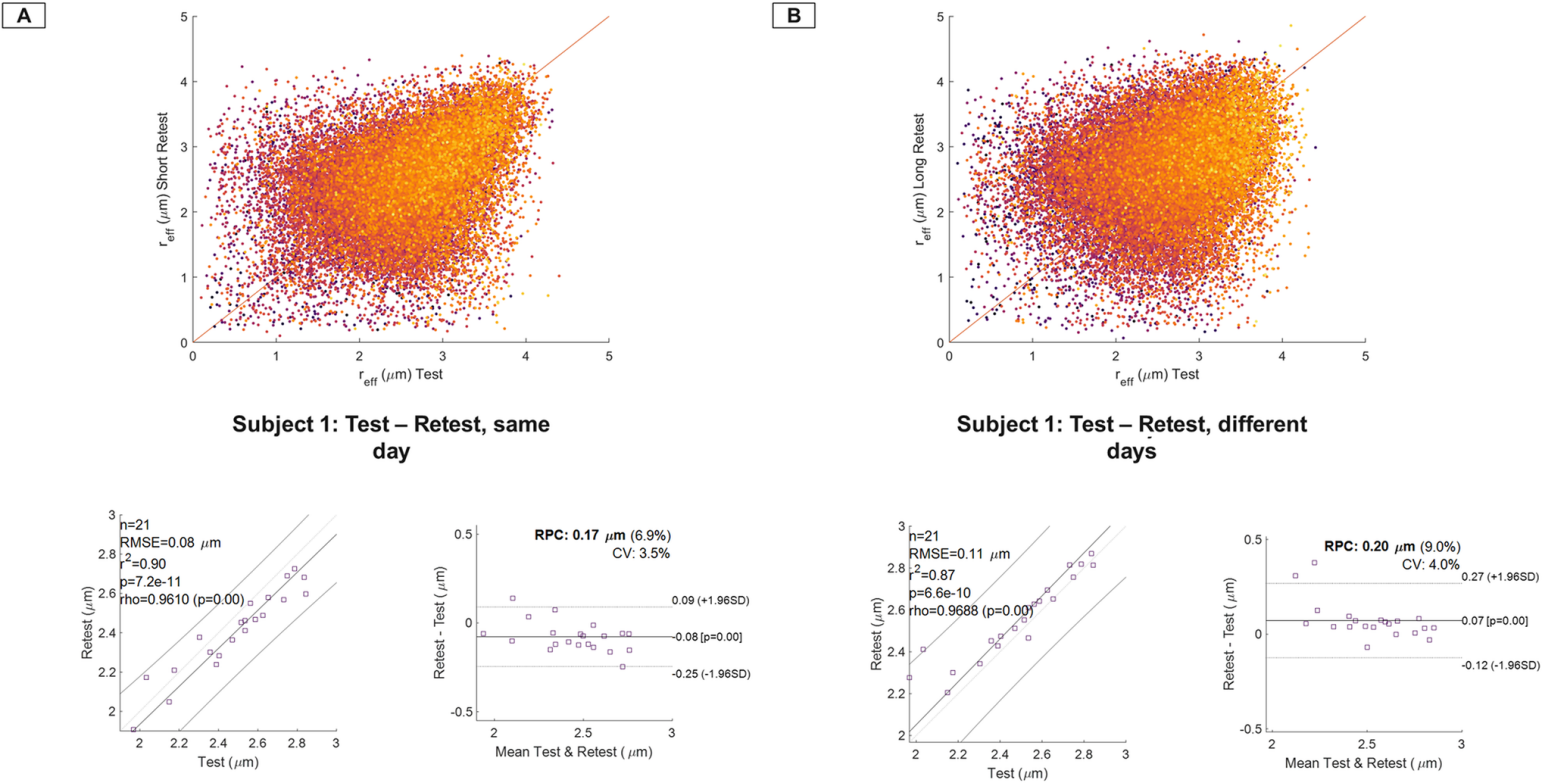
Kernel density plots (yellow is high density) for a representative test-retest volunteer for short (same scan day) and long (rescanned 7 days later) are shown in A and B for whole-brain white matter, highlighting strong correlation between the multiple scan sessions. Correlation and Bland-Altman plots are presented for 21 symmetric white matter parcels using the JHU-ICBM-81 atlas for the same subject for short-term and long-term repeatability analysis. Notably, high correlation was observed across all sessions with spearman correlation coefficient r = 0.95 and p-value at 0.00* (p-value is truncated), and mean coefficient of variation ≤4% across all parcels in this volunteer. Abbreviations: CV, coefficient of variation; RPC, reproducibility coefficient (1.96*SD).

**Fig. 8.**
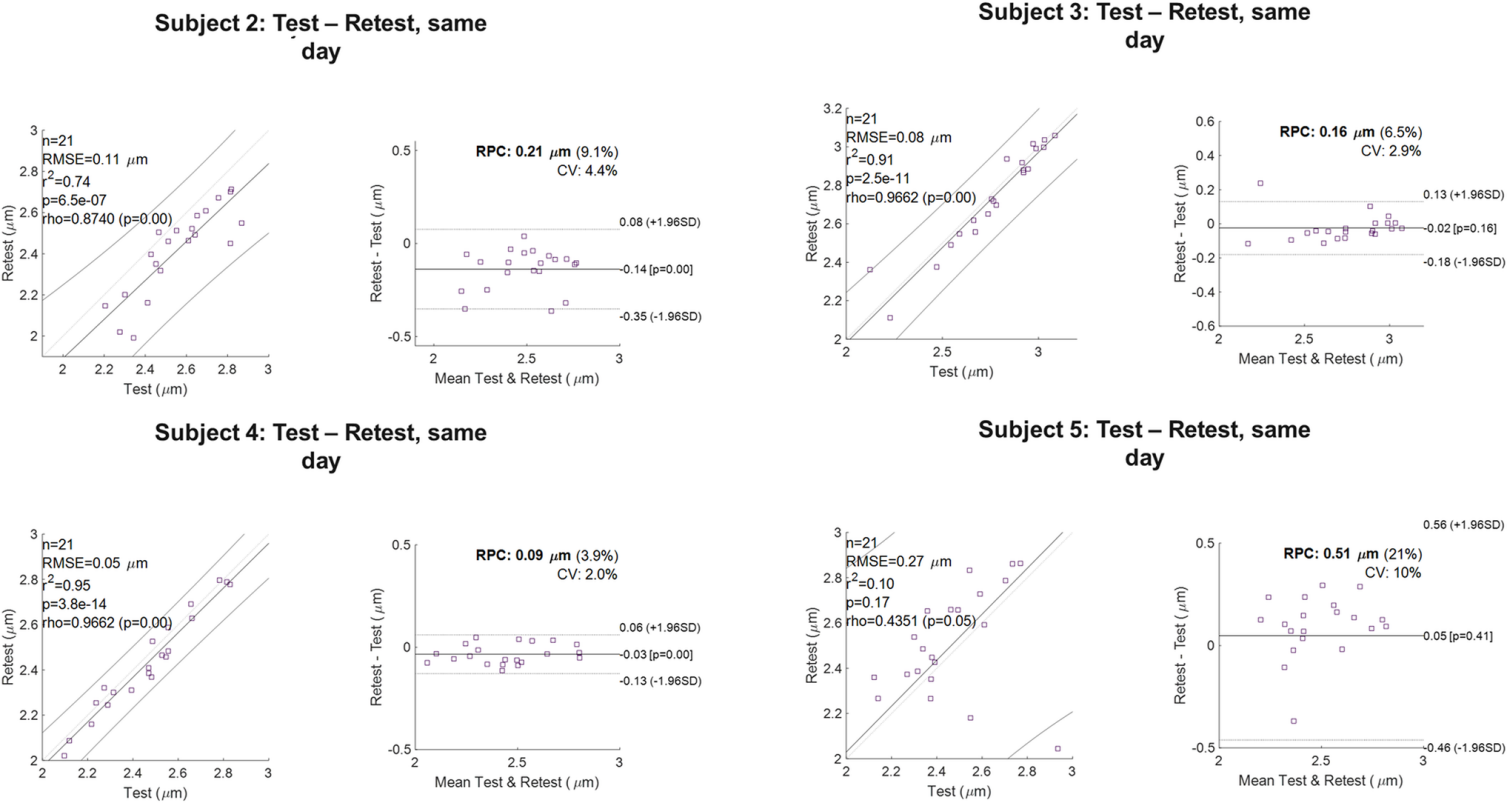
Bland-Altman analysis for 21 symmetric white matter parcels using the JHU-ICBM-81 atlas for test-retest over four volunteers is presented. The solid lines represent mean difference ± 1.96× standard deviations of the difference. Abbreviations: CV, coefficient of variation; RPC, reproducibility coefficient (1.96*SD). Correlation plots and Bland-Altman analysis for 21 white matter parcels in 4 test-retest volunteers, highlights lack of systematic bias and high reproducibility. On average, the coefficient of variation was 3.2%, with a reproducibility coefficient of 0.16 μm (6.6%). Abbreviations: CV, coefficient of variation; RPC, reproducibility coefficient (1.96*SD).

WM voxel-wise and JHU-ICBM-DTI-81 parcel-based test-retest repeatability of the *r_eff_* was evaluated on a per subject basis. Figures 7 and 8 highlight the correlation and Bland-Altman analysis, with the absolute mean difference and 95^th^ percentile confidence intervals, for short- and long-term test-retest. Kernel density plots for whole-brain WM (Fig. 7) underscore strong correlation for short (same scan session) and long-term (~7 days) test-retest. Bland-Altman analysis for short- and long-term test-retest show strong correlation and repeatability, with tighter variations in short- (CV~3.5%) and long-term test-retest (CV~4.0%). Spearman’s rank correlations coefficient on average showed high repeatability with positive correlation between test-retest, r = [0.95], p = 0.00* (p-value is truncated).

On average, across all subjects, the CoV for symmetric WM parcels was 3.2%, with a CoR of 0.16 μm (6.6%) (Fig. 8). Supplementary Table T1 tabulates the ICC across subject and parcel-wise test-re-test, which ranged from moderate to good (ranging between 0.67–0.97). This range of variation underscores that differences are dominated by physiological noise rather than systematic bias with the good-to-high reproducibility demonstrating the same order of magnitude as reported for conventional DTI and DKI.

## 4 Discussion and Conclusions

Quantitative features characterizing the intra-axonal space provide invaluable insight into the microstructural organization of the brain. In this study, the feasibility (by using a comprehensive high b-value diffusion dataset), and repeatability (by using a systematic test-retest paradigm) of utilizing MAGNUS for *in-vivo* non-invasive mapping of *r_eff_* is presented. Utilizing the full performance space for MAGNUS for *in-vivo* imaging, a simplified biophysical model-based approach was leveraged, which has previously been validated in pre-clinical and in whole-body high-performance MRI scanners (Assaf et al., 2008; Barazany et al., 2009; Horowitz et al., 2015; McNab et al., 2013; Veraart et al., 2020, 2021). Peripheral Nerve Stimulation (PNS) imposes constraints on the use of high gradient amplitudes and rapid slew rates in whole-body MR gradient coils, thereby limiting the ability to fully exploit the systems performance capabilities. However, head-only asymmetric high-performance systems reduce gradient-induced electric fields quadratically from isocenter, and provide imaging advantages with respect to full-brain coverage, improved image quality with high SNR and low distortion in addition to utilization of the expanded imaging parameter space. Our work highlights this practical platform for advancing neuroimaging in a way that substantially mitigates PNS constraints from previous studies.

The data highlight distinct patterns of *r_eff_* distribution in the white matter of healthy volunteers. Mean distribution in whole-brain white matter, and coarse segments over select white matter parcels were in good-to-excellent agreement with microstructure trends reported in prior *in-vivo* MR literature from other studies which used the biophysical model (Veraart et al., 2020, 2021), Axonal Diameter Index (Fan et al., 2020), and AxCaliber (Assaf et al., 2008) (Supplementary Fig. S5). Despite the different length scales and confounds from fixative and histological sample preparation, histology has reported *r_eff_* distributions ranging from 0.54 μm to large axons >3 μm (<1%) (Caminiti et al., 2009). These distinct, yet complementary approaches converge to strikingly similar estimates and distributions of effective intra-axonal radii in the *in-vivo* human brain observed with MRI. Figure 6 and Supplementary Figure S5 show the individual mean *r_eff_* from all volunteers in this study along with those reported in literature.

Importantly, this study highlights the topological hemispheric differences in brain *r_eff_* as a function of white-matter asymmetry (Fig. 6). Averaged over the entire study group, left and right dominated asymmetries were evident in specific white matter parcels. Several studies have highlighted functional lateralization as a conserved feature of the CNS with underlying microstructural substrates (Bianco et al., 2008; Honnedevasthana Arun et al., 2021; Korbmacher et al., 2024). Previously, Veraart et al. (2020) had commented on visually observed hemispheric asymmetry in their data, albeit with a different MR scanner configuration, study design and population. Unsurprisingly, subject-specific variations drive the difference—with parcels such as the uncinate (involved with mnemonic associations), cingulum bundles (memory consolidation), and the peduncles (motor task) highlighting the largest deviation between the hemispheres across the study participants. Though, in this feasibility study—with a limited sample size—covariates for age, sex, and handedness were not introduced, hemispheric differentiation provides a critical pathway to complement understanding on how brain asymmetries impact normative studies. The underlying left-right asymmetries within neural circuity, reported in this study, are reported to play a central role in cognitive and behavioral neurology and can be used to provide critical information on how individual differences contribute to functional pathology. The relevance of these findings would benefit from a larger diverse and representative population study to contextualize the impacts of learning, plasticity, and pathology.

It is acknowledged, however, that MR measurements are limited by practical limits of detection and are inherently tail-weighted approaches, where confounds such as longer gradient pulse-widths, partial volume, distribution of axons in the voxel being represented by a single metric, and not taking axonal caliber variation or undulations into account (H. H. Lee et al., 2020; Nilsson et al., 2012), or the presence of non-exchanging compartments such as soma (Palombo et al., 2020) for a realistic model-based approach limit precise interpretations. As shown, theoretically, and in the simulations, the log(S) representation gives rise to an $r^{4}$ dependence. To achieve closer agreement with histology, even higher gradient performance in MR neuroimaging hardware may be required. However, even when tailoring the experiment to utilize the strongest diffusion encoding gradient strength available, there was a lower bound on the intra-axonal effective radius that could be detected robustly. As shown by the simulations, as the *G*_max_ (system performance) was increased from 200 mT/m to 300 mT/m, the probability distribution shifts to the left, highlighting increased sensitivity to relatively smaller axons as diffusion encoding pulse widths (δ), mixing times (△), and TE are reduced, indicating that this maybe further improved through ongoing efforts to increase system performance for *in vivo* human MR scanning (Fig. 3). A key caveat, however, is that PNS imposes limitations, and simply increasing gradient strength beyond 300–400 mT/m is not sufficient without taking into consideration significant gradient redesign. As gradient amplitude increases, PNS imposes constraints on the gradient pulse rise times, diminishing additional gains in reductions of δ, △, and TE (Supplementary Fig. S8). Any further benefit has to be realized by either re-architecting the gradient driver, redesign the gradient coil to be more or less asymmetric albeit at the cost of decreasing efficiency, compactness of design, and introduction of winding loops at patient end (Lee & Bernstein, 2022; S. K. Lee et al., 2016); or by compromising the design such that it does not address PNS constraints at all scan positions (Davids et al., 2023, 2024).

Additionally, in this study, a framework for increased precision in mapping voxel-wise contribution is presented, by integrating real-valued diffusion (RVD) data handling and gradient non-linearity correction to reduce measurement bias from noise and system effects. A few studies have highlighted the benefit of RVD data for model fitting and signal processing in the clinical setting for robust parameter estimation (Prah et al., 2010; Sprenger et al., 2017). As shown in Supplementary Figure S4 and in simulations by Fan et al. (2020), intra-axonal radius estimates highlight a shift in mean histograms to the resolvable range with real-valued diffusion compared to magnitude-based approaches. As higher diffusion weighting is explored in conjunction with increased spatial resolution, SNR losses increase due to the increasing the footprint of correlated rectified noise. Here, real-valued approaches, cognizant of the propagation of noise through the experiment and reconstruction, stand to benefit.

*In-vivo* test-retest variability is governed by thermal noise, and intra- and inter-subject variations. However, test-retest statistics across the volunteers showed good-to-high repeatability on voxel-wise basis and in white matter parcels, with single unit coefficient of variation values demonstrating the ability to detect subtle changes in the brain on a per-subject basis. Both CoV (Figs. 7 and 8) and ICC (Supplementary Table T1) were used to assess variability within the datasets and establish the floor of change in the brain that can be consistently/reliably observed (outside of random variation or noise) in studies leveraging effective intra-axonal radii for biomarker evaluation or neuroplastic changes.

Intra-axonal distributions provide key insights into structural substrates for functional measures. The simplified biophysical space available with ultra-high b-value diffusion imaging has identified a possible new imaging biomarker with potentially high clinical value. As shown in this study, even with abbreviated protocols of ≤21 min, the technique offers discernable new contrast mechanisms in pathologies not observed via conventional imaging. Axonal radii/diameters are directly related to conduction velocity: the largest axons are the fastest axons, and any modification or alterations to this space can provide early differentiators for disease onset and progression. In addition, *r_eff_* have high utility in brain connectivity and identifying plastic processes as a function of learning and memory consolidation, thereby providing insight into how the brain learns and allocates information. Our results further establish a lower bound for detecting the changes in *r_eff_* in response to learning or intervention, an important component for assessing microstructural changes, such as for neuroplasticity, Alzheimer’s Disease, and neuropsychiatric disorders, for treatment monitoring.

## Disclaimer

The opinions or assertions contained herein are the views of the authors and are not to be construed as the views of the U.S. Department of Defense, Walter Reed National Military Medical Center, or the Uniformed Services University.

## Supplementary Material

Supplementary Material

## Data Availability

Data available subject to successful conclusion of a data-sharing agreement.
