# Supplementary material for: Feasibility of brain intra-axonal microstructure imaging with ultrahigh B-encoding using MAGNUS ultra-high-performance gradients
Source: Imaging Neurosci (Camb). 2025 Jul 17;3:IMAG.a.68. doi: 10.1162/IMAG.a.68 (PMC12330845; doi:10.1162/IMAG.a.68)

Supplementary Figures and Supplementary Figure Captions

Supplementary Table T1. The IntraClass Correlation (ICC) for whole brain white matter across all subjects and individual test-retest is tabulated for the five volunteers recruited in this study. ICC estimates were interpreted based on the guidelines: <0.5 poor reliability, 0.5≥x≥0.75 moderate reliability, and >0.75 indicate good reliability. ICC values were interpreted considering the 95% confidence interval.

	ICC	Lower bound	Upper bound
Whole brain white matter - All subjects	0.622	0.470	0.783
Subject 1 test-retest	0.885	0.742	0.951
Subject 2 test-retest	0.669	0.351	0.850
Subject 3 test-retest	0.965	0.918	0.986
Subject 4 test-retest	0.859	0.690	0.940
Subject 5 test-retest	0.830	0.633	0.927

Supplementary Figure S1. Spherical mean magnitude and real valued diffusion data highlights the superposition of noise floor, and the absence thereof with decorrelated phase filtering. The span of application b-values highlights the benefit not only over high b-values but also in signal conspicuity in the low to medium b-value range for MAGNUS (>8 ms/μm^2). Sagittal reformats highlight the SNR gain even over T_{2w}/b=0 data for both magnitude and RVD.

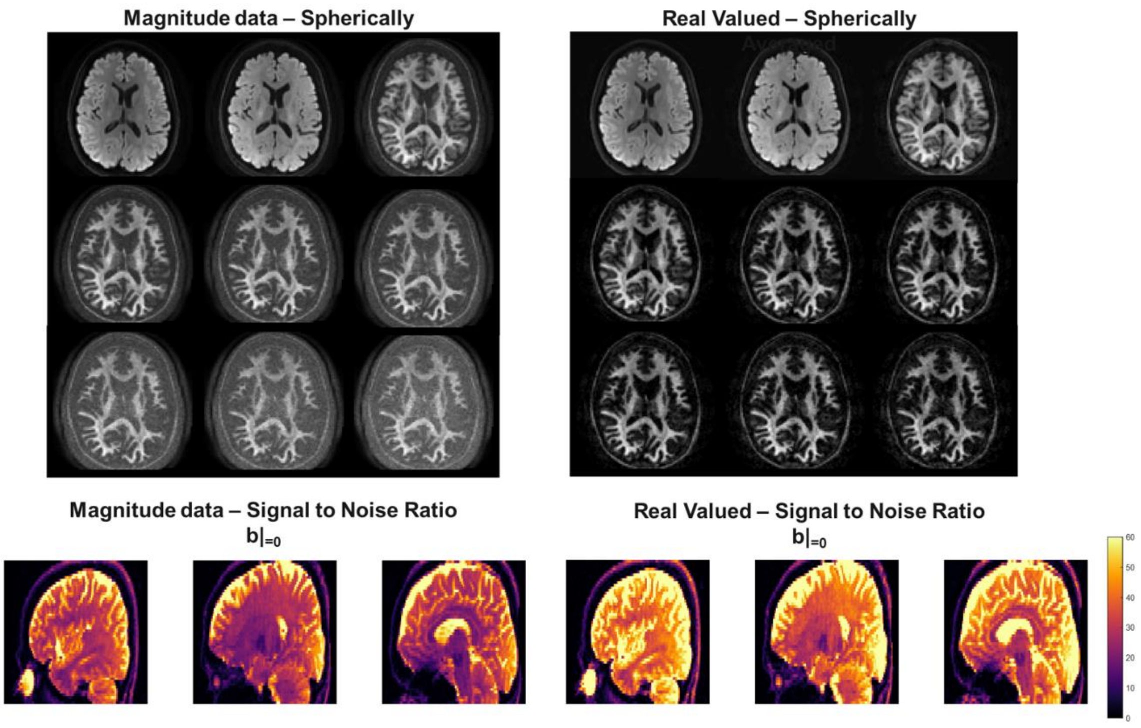

Supplementary Figure S2. B-value deviation maps (without any corrections) from one of the volunteers is presented to highlight gradient non-linearity impacts over a typical volunteer scan session using a dMRI protocol on the MAGNUS scanner with different gradient axis for diffusion encoding. At the edge of the scan FOV, non-linear effects are more sever and reach >20% from nominal prescribed values.

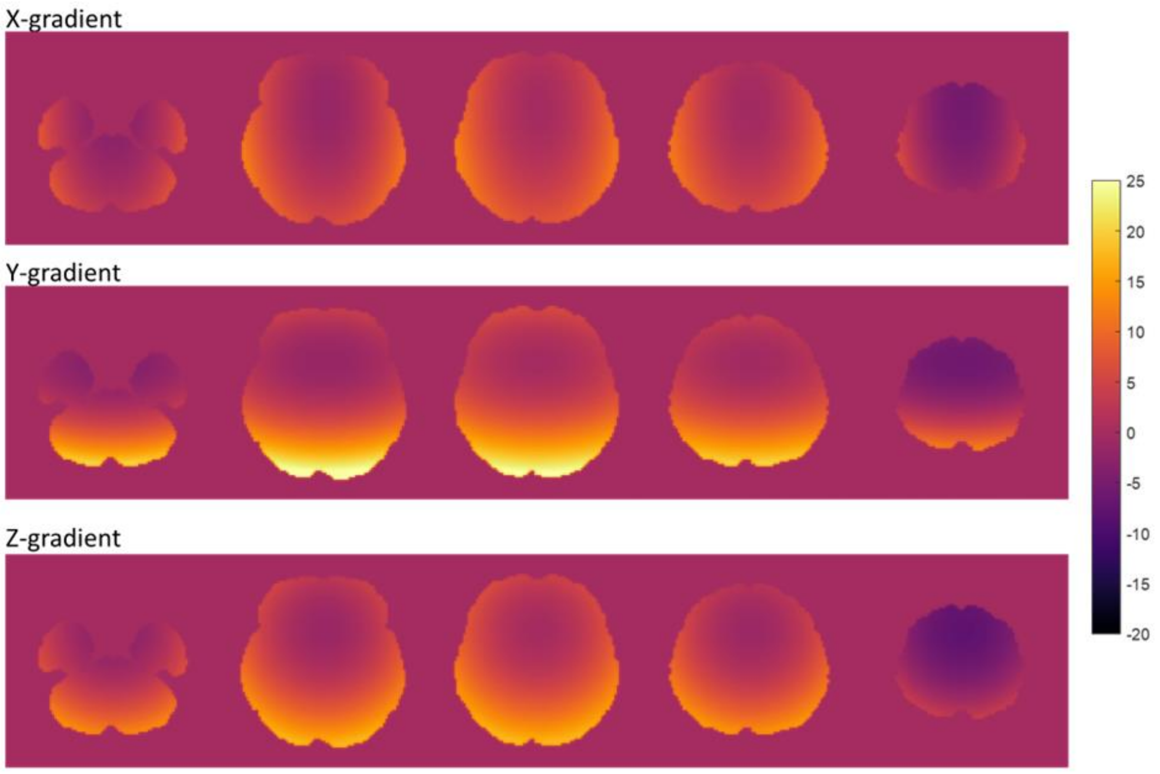

Supplementary Figure S3. Representative deviation map over a 26 cm FOV highlights the impact on applied gradient vectors as a function of position. At isocenter the applied vectors (red markers on sphere) and measured vector (green markers on sphere) are identical (overlaid). A linear translation in SI highlights only scaling on the applied vector and no rotation. Diagonal and off-diagonal translation highlight both a rotation in the b-vector as well as a scaling on the b-value.

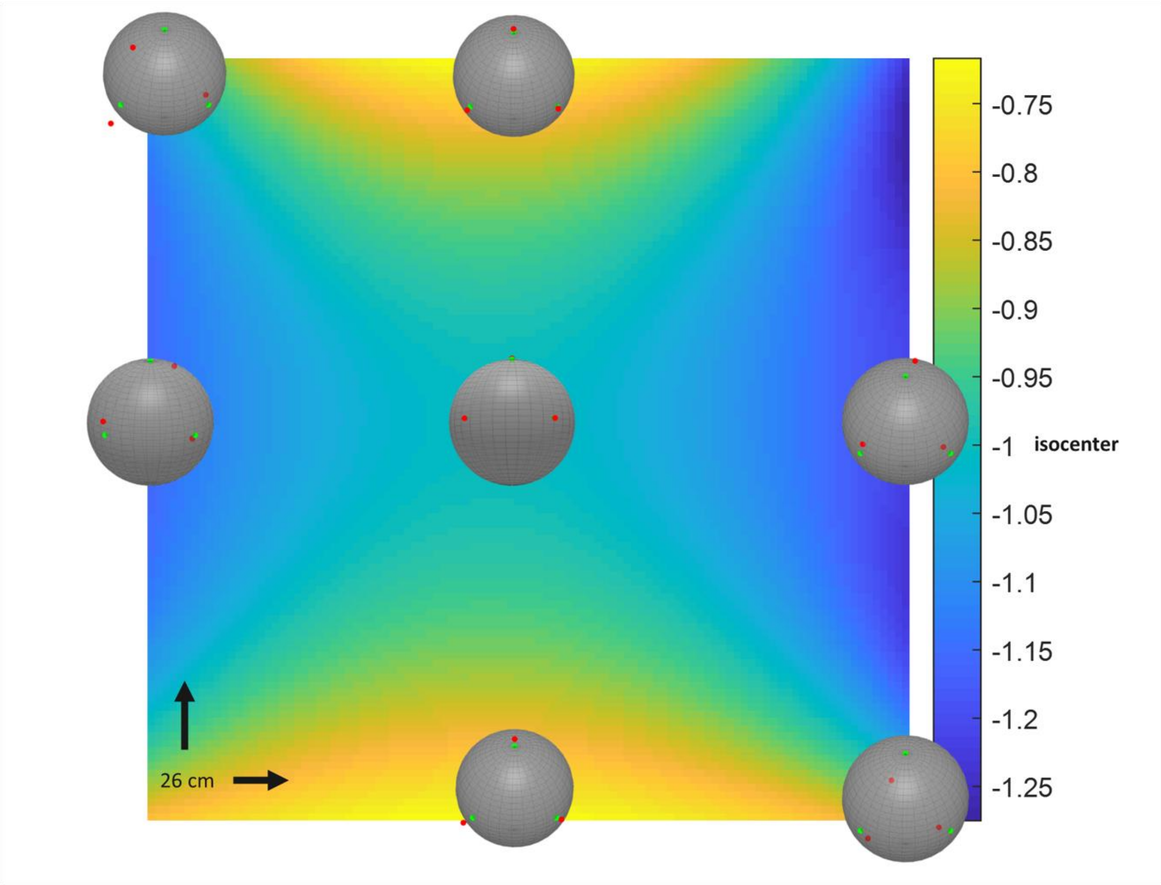

Supplementary Figure S4. Differences between magnitude and real-valued data processing highlights the bias in signal attenuation in high-b values and the difference in fit estimates. For magnitude data, fast non-local means denoising was used coupled with Koay and Bassers method of moments for Rician estimation. Real valued data was processed as defined in the methods section of this manuscript using decorrelated phase filtering.

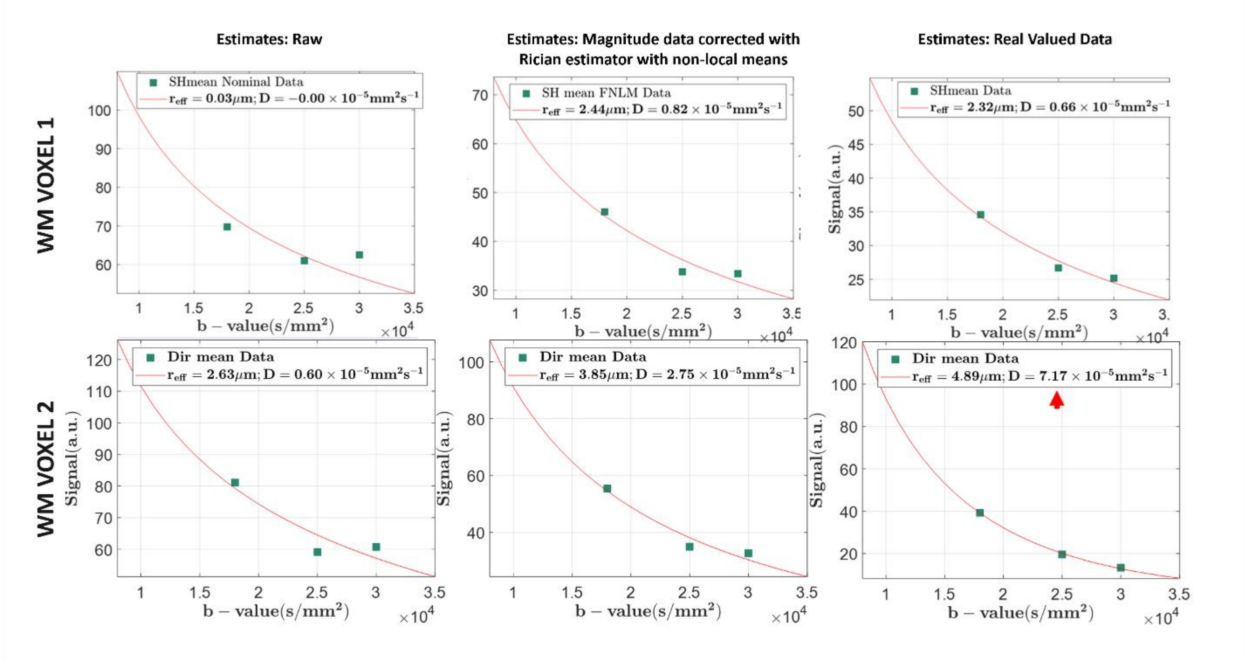

Supplementary Figure S5. Histogram for whole brain white matter intra-axonal radius is presented with line plots (black solid line) highlighting mean effective intra-axonal radii from all volunteers in this study. Mean values show good-to-excellent correspondence for both the abbreviated and expanded diffusion protocols. Red-dotted lines show correspondence with mean intra-axonal effective radii reported in prior literature.

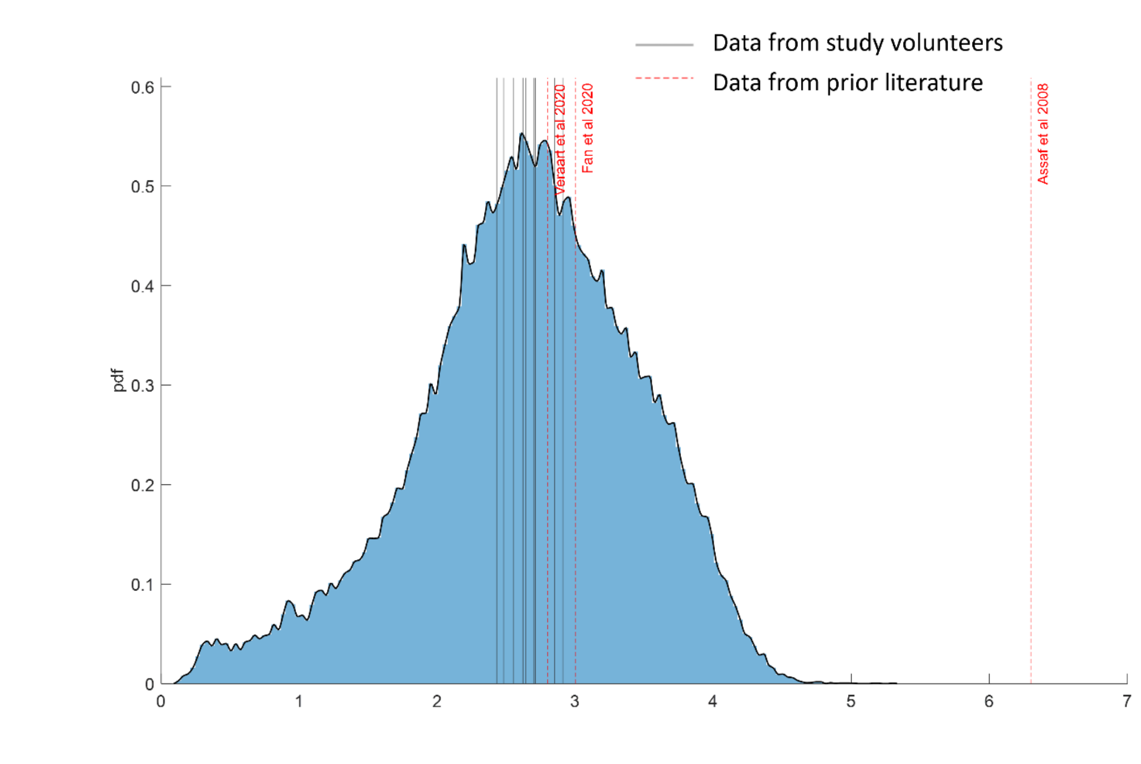

Supplementary Figure S6. Representative maps highlight directionally averaged (A) Magnitude (no denoising) and (B) decorrelated phase corrected real-valued data. (C) Imaginary residuals largely highlight absence of anatomy, indicating noise rather than structure has been reduced. Note: Images are not uniformly scaled.

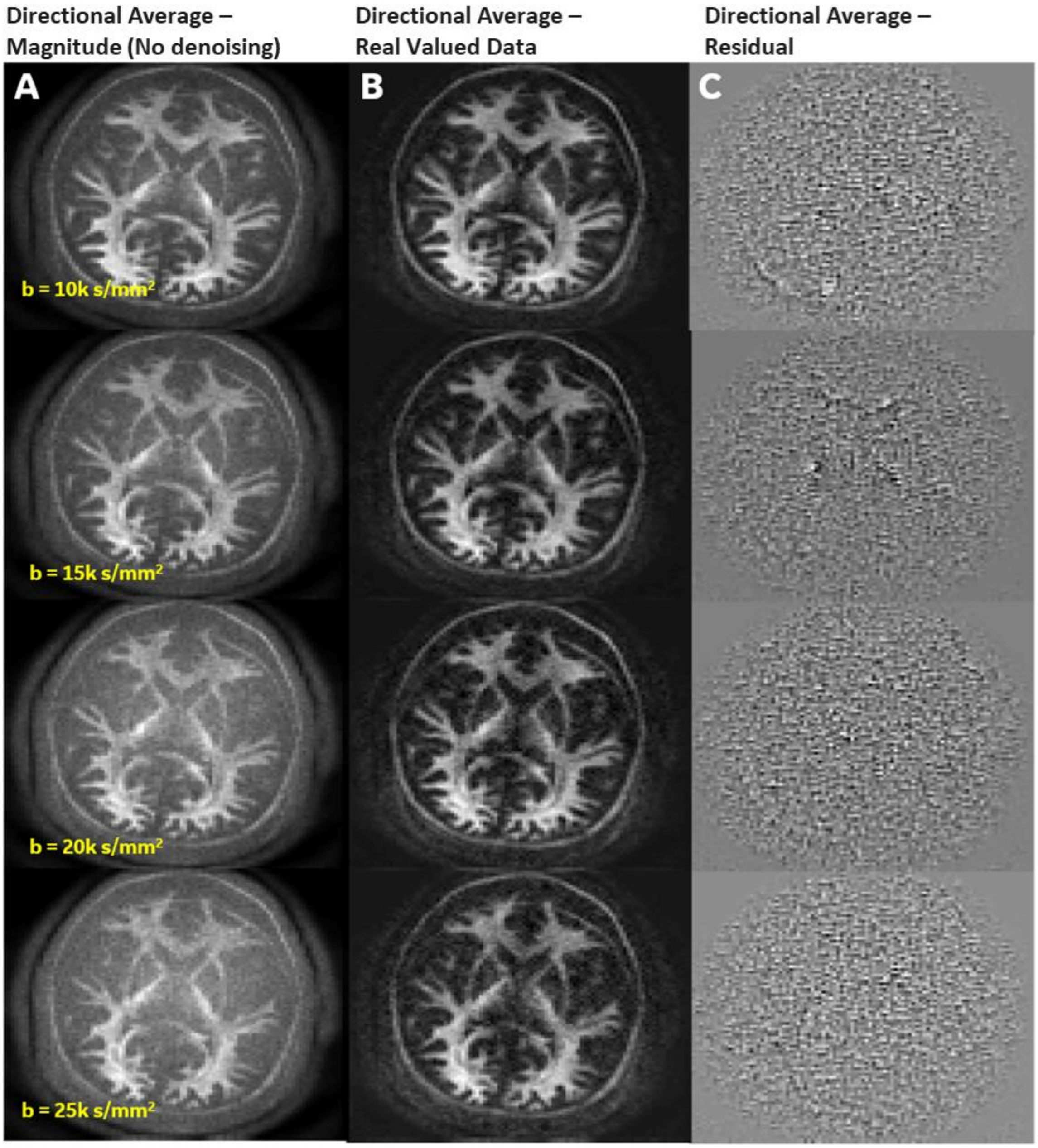

Supplementary Figure S7. Echo planar imaging readout waveforms at two slew rates, 200 T/m/s (clinical whole body scanner performance), and 500 T/m/s (high performance MRI gradient). As compared, the whole-body gradient performance parameters result in longer echo spacing and echo train length durations. The high-performance gradient images show reduced signal 'pile-up', phase aliasing and reduced image distortion.

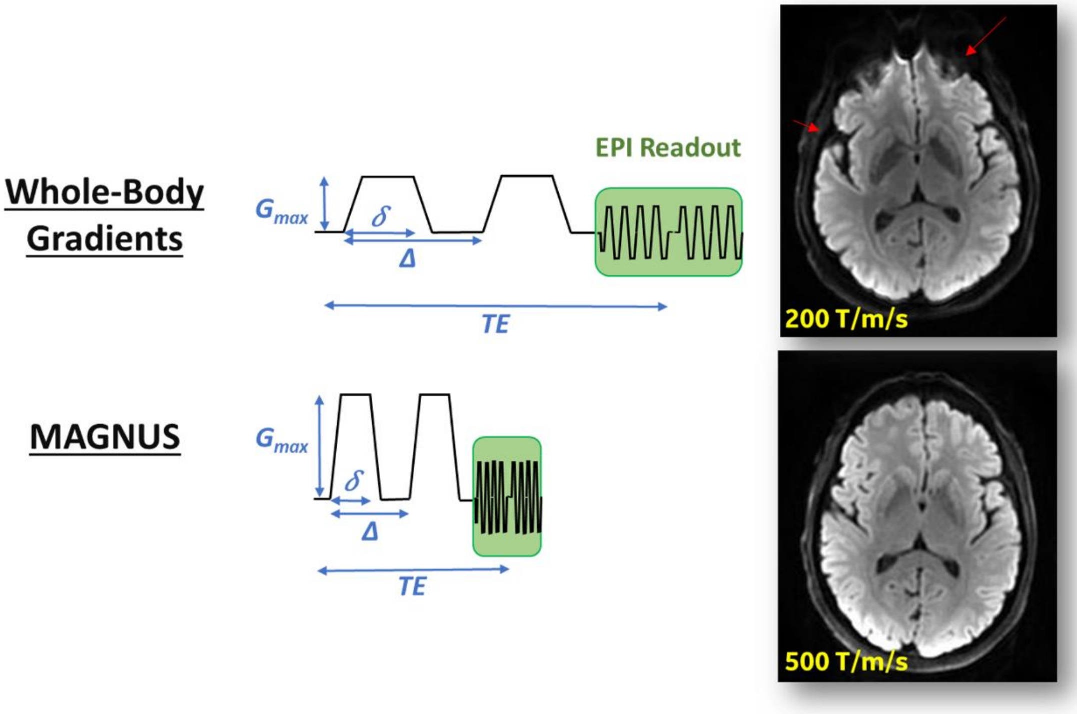

Supplementary Figure S8. dMRI acquisition space simulations highlight the relationship between diffusion encoding with a PGSE implementation and associated echo time reduction as a function of gradient coil design and gradient driver performance (driver performance simulated from 1 MVA to 4 MVA using the same MAGNUS gradient coil design). However, it is noted that past a certain point, without gradient redesign any further advantages are limited by human peripheral nerve stimulation thresholds.

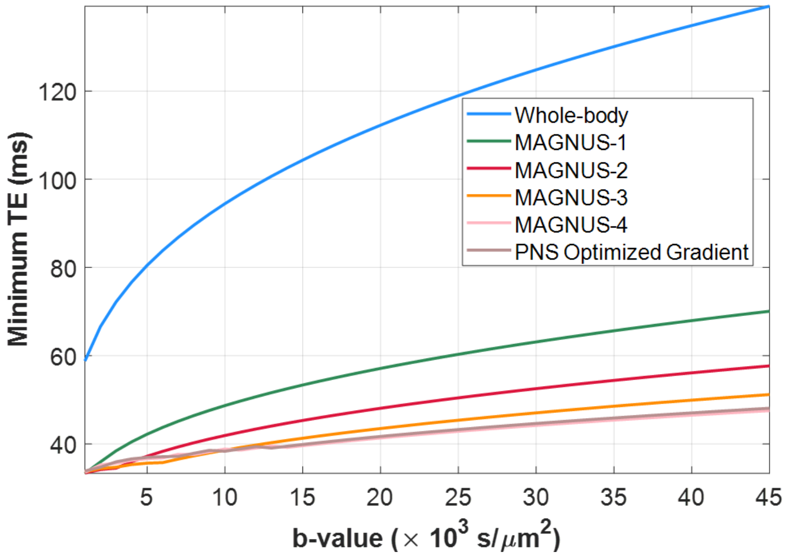

Supplement: Supplementary Material [file IMAG.a.68_supp.pdf]
